# Identifying facilitators and barriers for adolescents participating in a school-based HIIT intervention: the eXercise for asthma with commando Joe’s® (X4ACJ) programme

**DOI:** 10.1186/s12889-020-08740-3

**Published:** 2020-05-01

**Authors:** Catherine A. Sharp, Melitta A. McNarry, William T. B. Eddolls, Harriet Koorts, Charles O. N. Winn, Kelly A. Mackintosh

**Affiliations:** 1 Public Health Collaborating Unit, School of Health Sciences, Bangor University, Bangor, UK; 2 Applied Sports Science Technology, Exercise and Medicine (A-STEM) Research Centre, College of Engineering, Bay Campus, Swansea University, Swansea, UK; 3 Institute for Physical Activity and Nutrition (IPAN), School of Exercise and Nutrition Sciences, Deakin University, Geelong, Australia

**Keywords:** High-intensity interval training, School, Focus groups, Non-attendee, Discipline, Benefits, Enjoyment

## Abstract

**Background:**

High-intensity interval training (HIIT) elicits numerous health benefits, but little evidence is available regarding the feasibility of delivering school-based HIIT interventions. The aim of this study was to explore adolescents’ perceptions of a 6-month, 3 × 30-min sessions per week, HIIT intervention delivered either before or after school.

**Method:**

Eighty adolescents allocated to the intervention group (13.3 ± 1.0 years; 45 boys) were invited to take part in semi-structured focus groups post-intervention. Participants were categorised as attendees (≥40% attendance) or non-attendees (< 5% attendance). Data were transcribed verbatim and thematically analysed deductively, with key emergent themes represented using pen profiles.

**Results:**

Results showed that a school-based HIIT intervention can be an enjoyable form of exercise. Irrespective of attendance, similar facilitators and barriers to participating were highlighted, including benefits of participation, content of the exercise session and the intervention instructor.

**Conclusion:**

This study provides support for the delivery of a HIIT intervention in a school setting but highlights the importance of a flexible design and delivery to accommodate competing interests. There is a need to educate adolescents on the possible benefits of participation and to make the sessions enjoyable in order to increase their extrinsic and intrinsic motivation to sustain participation.

## Background

Health and social benefits of engaging in physical activity across the lifespan are well-documented globally [1, 2]. Nonetheless, a disconnect remains between the recognised benefits of physical activity and the proportion of individuals who achieve the recommended physical activity guidelines [3]. Indeed, worldwide, approximately four in five adolescents (81%) are not meeting the global physical activity recommendations for optimal health [4]. The recommendation for adolescents is 60 min moderate-to-vigorous-intensity activity [5], on average, every day. Consequently, in 2018 the World Health Organization set a target of reducing physical inactivity by 15% by 2030 [6]. The challenge, however, is creating sustainable and economic methods of increasing physical activity levels.

Exercise, a sub-component of physical activity, which involves structured, planned and repetitive movements [6], has been identified as an effective method of increasing physical activity and improving health outcomes such as blood pressure and cardiovascular risk factors in adolescents [7, 8]. A specific form of exercise that has gained popularity in recent years is high-intensity interval training (HIIT), which is characterised by “brief, repeated bursts of relatively intensive exercise separated by periods of rest or low-intensity exercise” ([9]; pg 406). However, the effectiveness of HIIT interventions to improve health outcomes for adolescents, especially body composition and blood pressure, remains equivocal [10].

The short time requirement for HIIT is often what appeals to individuals [11]. However, some interventions have demonstrated adolescents only attend around half of the available HIIT sessions [12]. In order to increase adolescents’ rate of attendance, and thereby maximise the impact of such interventions, it is important to understand adolescents’ attitudes towards their design and evaluation [13]. One framework that may aid in this is the Youth Physical Activity Promotion Model (YPAMA [14];). This model provides a broad perspective to help understand variables that support and challenge adolescents’ physical activity. It is well recognised that attitudes, self-efficacy and social norms (either from peers or caregivers) predict behaviour (Theory of Planned Behaviour [15];), including physical activity behaviour [16]. In order to improve attitudes and self-efficacy in the target audience to increase physical activity levels, it is essential to co-create interventions [17] and to treat adolescents as experts in the process.

Process evaluations are a method of assessing whether programme activities have been implemented as intended [18]. Whilst such evaluations are regularly conducted on school-based physical activity interventions, only a small number have been conducted specifically on school-based HIIT interventions. In 2015, Costigan et al. [19] reported that adolescents attended an average of 2.2 out of 3 sessions per week over the course of an 8-week HIIT intervention when delivered during lunch time. Furthermore, Buchan et al. [20] noted that even though adolescents initially lacked confidence, their confidence grew over the course of a 7-week HIIT intervention, with key reasons for continuing their participation being “participant support, competition and not letting anyone down” (pg 8). Congruent with Costigan et al. [19], the average attendance for the intervention in Buchan et al. [20] was also 2.2 out of 3 sessions, though the timing of when the intervention was delivered was unclear. Moreover, Leahy et al. [12] implemented a 14-week HIIT intervention consisting of two teacher-delivered and one student-led self-directed session; parents were also sent an information video with guidance on how their child could continue to complete the prescribed three sessions a week during holiday periods. Indeed, Leahy and colleagues [12] found an average attendance of 1.9 out of 3 sessions during school weeks, which decreased to 1.7 when including school holiday weeks. More specifically, the majority of adolescents enjoyed the practical HIIT sessions, with improving health, or forgetting to do the HIIT session, being the most common motivator and barrier to participation, respectively. Elsewhere, adolescents reported enjoying taking part in three laboratory-based HIIT sessions, more than moderate-intensity interval training [21]. Whilst it is evident that adolescents have an appetite for participating in HIIT interventions, their average attendance decreases over time, thus impacting on intervention effectiveness. Moreover, to date, only short-to-medium length HIIT interventions (i.e., 3-weeks to 3-months) delivered during the school day have been evaluated and participants have not been categorised based on their level of attendance [10].

The *eXercise for Asthma with Commando Joe’s® (X4ACJ)* HIIT programme was implemented in 2015/16 in secondary schools across South Wales by an ex-military fitness instructor. The 6-month HIIT intervention involved 3 × 30-min sessions per week, was delivered either before or after school [22]. Whilst the intervention yielded significant improvements in adolescents’ cardiorespiratory fitness and maintained body mass index at the 6-month follow-up, it did not improve lung function, asthma control or quality of life [22], the reasons for which remain to be elucidated. Therefore, the main aim of this study was to understand the perceived facilitators and barriers to participating in the X4ACJ HIIT programme, exploring differences based on attendance rates.

## Method

### Participants

A sub-sample of eighty adolescents (13.1 ± 1.0 years; 45 boys) across school years 7–10 (11–15 years) from a single secondary school in South Wales, UK, were recruited to participate in focus groups based on their participation and attendance in the intervention group of the X4ACJ programme. The school, encompassing approximately 900 children, was situated in a more affluent urban area (quintile 4) based on WIMD (2019), and 18% of the total school pupils had free school meals as reported by Estyn in 2013. Informed signed consent to participate in the focus groups was obtained prior to the intervention commencing, with participants subsequently invited by a stratified approach, depending on their attendance. This sample represents 36.2% of the total intervention group sample and has a similar sex distribution as achieved in the quantitative measures (24; 56.3% versus 52.5% boys, respectively). Participants who actively engaged and attended 40% or more of the X4ACJ programme sessions were assigned to the ‘attendee group’ (*n* = 36; 23 boys), and those who attended less than 5% of the intervention sessions were assigned to the ‘non-attendee group’ (*n* = 44; 22 boys). Ethical approval was granted by Swansea University A-STEM Ethics Committee (PG/2014/29). Written informed parental and head teacher consent were obtained at the outset of the study and adolescent assent was obtained for each phase of the study.

### Procedures

Co-author (WTBE) facilitated 17 semi-structured focus groups, with 3–6 adolescents in each group, to ensure that the groups were easily controllable but sufficiently lively to derive insightful feedback [23]. For pragmatic reasons, one session consisted of only two participants; no differences between emergent themes were detected and therefore it was deemed appropriate for inclusion. Participants were assigned to focus group sessions based on attendance and further stratified by school year. The sessions were conducted in a quiet space within the school environment and lasted 45 ± 7 min. To enable social interaction and observer involvement, participants were positioned around a circular table with the researcher seated amongst the participants [24]. An ice-breaker was used at the start of the session to help familiarise the participants with the environment.

Age-appropriate focus group questions were structured to elicit an understanding of the participants’ perceptions of the intervention (Table 1). The questions focused on five key areas: session content, attendance, HIIT, benefits of participation, and the instructor. All focus groups were digitally audio-recorded (Samsung Galaxy S7 Edge, Suswon, South Korea).

**Table 1:** Example focus group questions for adolescents in years 7–10 (11–14 years)

Section	Example questions
Exercise session	What was your least favourite session and why?
	What could we have changed about the sessions to make them more enjoyable?
Attendance	What made you want to keep coming to sessions?
	How did your impression of the sessions change over time?
HIIT	What did you most enjoy about HIIT?
	What did you least enjoy about HIIT?
Benefits	What, if any, do you feel the benefits of attending are?
	Do you think you have had any personal benefits from the sessions? What were they?
Instructor	What did you think of the Commando Joe’s instructor?
	How do you think the instructor delivered the sessions?

### Data analysis

Data was transcribed verbatim with subsequent immersion for data familiarisation by WTBE. Using data coding and identification of themes by attendance group, transcripts were thematically analysed by WTBE [25]. Data was initially coded deductively, categorising the data using elements of the YPAPM and broader literature-informed pre-hypothesised concepts (e.g., exercise session, benefits, instructor [26];). Thereafter, the categorised data were coded inductively based on smaller, more specific, observations from the data (e.g., motivation, competition, group size and competing interests). Once all the data were coded, significant broader patterns were clustered into overarching themes. All themes were reviewed by WTBE to ensure they were consistent with the narrative of the transcribed data. Succinct and informative theme names were defined and presented using pen profiles.

Pen profiling is a method of presenting data in an intelligible manner for both quantitative and qualitative researchers (see [27, 28]). In line with recent qualitative research [29], methodological rigour of the thematic analysis was ensured using the ‘critical friends’ approach [30]. Specifically, the pen profiles were constructed by WTBE and presented to the last author (KAM), an experienced qualitative researcher, who acted as a theoretical sounding board. During this process, KAM independently read the transcripts and subsequently challenged WTBE’s analytical decisions regarding the themes presented in the pen profiles, encouraging reflection upon the interpretation of the data. Following agreement, co-author MAM then separately critiqued and challenged the pen profiles in reverse, back to the transcripts, until a consensus was reached, and the pen profiles were finalised.

## Results

**Fig. 1:**
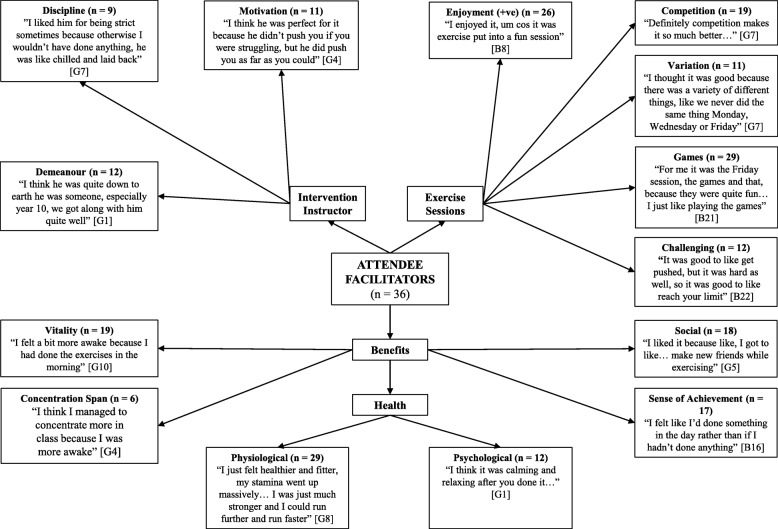
Attendee facilitators - pen profile of perceptions of a 6-month high-intensity interval training intervention. B = boy, G = girl, +ve = positive

**Fig. 2:**
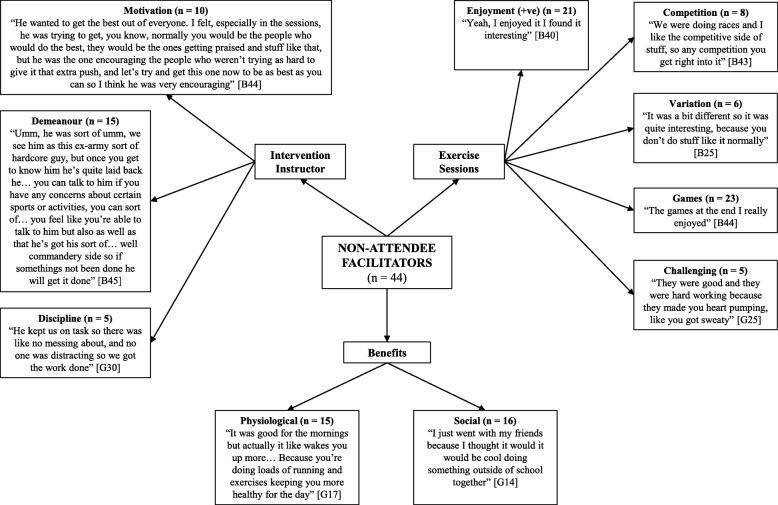
Non-attendee facilitators - pen profile of perceptions of a 6-month high-intensity interval training intervention. B = boy, G = girl, +ve = positive

**Fig. 3:**
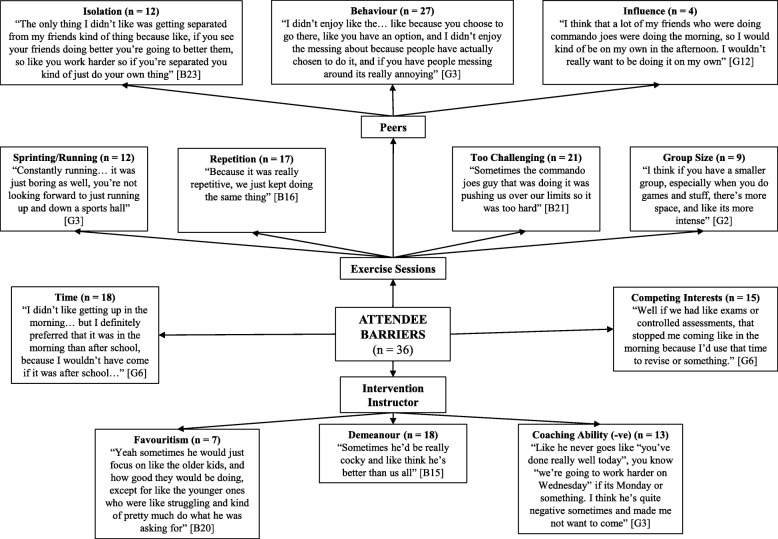
Attendee barriers - pen profile of perceptions of a 6-month high-intensity interval training intervention. B = boy, G = girl, -ve = negative

**Fig. 4:**
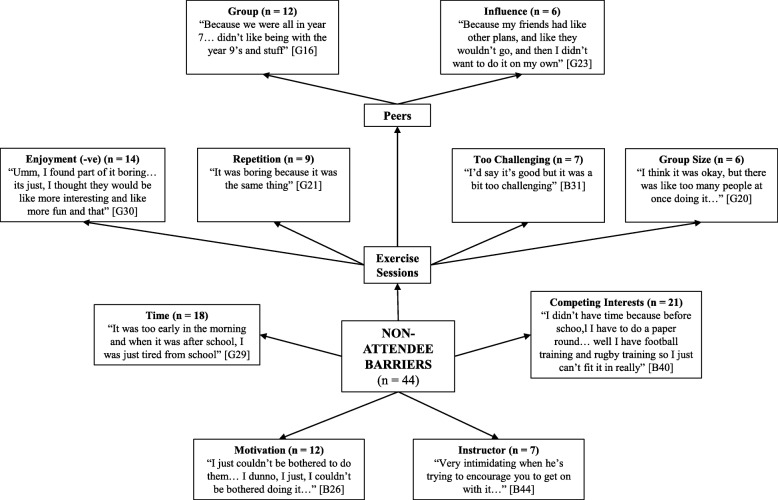
Non-attendee barriers - pen profile of perceptions of a 6-month high-intensity interval training intervention. B = boy, G = girl, −ve = negative

Four pen profiles were constructed to represent the facilitators and barriers to participating in, and the positive and negative perceptions of, the intervention for the attendee group and non-attendee group (see Figs. 1, 2, 3 and 4). Irrespective of attendee group, the same three key emergent themes were derived, namely: i) *benefits of participation*; ii) *content of the exercise sessions*; and iii) the *intervention instructor*. A key sub-theme within the benefits of participation raised by those in the attendee group was *health benefits*, whilst both groups highlighted *peers* as a potential barrier to participation. Quotes are denoted as A or NA for attendee and non-attendee, respectively, and B or G to represent boy or girl, respectively.

### Benefits of participation

The physiological benefits were identified as a driving factor for attendance in the majority of attendees (81%), compared to a minority of non-attendee group (31%): “*For me it was to get fitter…*” (A:B21) and “*I liked the exercising part to keep fit…*” (NA:G35). Participants in both groups identified the social benefits of attending, although the theme was more common in the attendee group (50% vs. 36%): “*I enjoyed… having fun with everyone*” (A:B3) and “*all my friends were doing it*” (NA:G18). Only the attendee group identified potential psychological benefits (33%): “*it made me feel better about myself*” (A:B23), with nearly half of the attendee group (47%) also noting an increased sense of achievement: “*It made me feel better about myself because it felt like I’d achieved something*” (A:B23). The attendee group expressed that taking part had a positive impact on their school life, for example, a participant reported it helped her “*get ready for work*” (A:G7). Finally, participants believed that the sessions improved their vitality (52%) and concentration spans (17%): “*pumped me up for the day as well then, like if I didn’t go to that in the morning, I would be exhausted in lessons all day*” (A:B5).

### Content of the exercise session

Five facilitators to attendance related to the exercise session content itself were described by both groups: i) *game features of the session*; ii) *enjoyment experienced during the session*; iii) *competitive aspects of the session*; iv) *the challenging nature of the exercise*; and v) *the variation of the session content*. These facilitators were less common in the non-attendee group. Both groups reflected on four barriers: i) *repetition of the session content*; ii) *the session being too challenging*; iii) *group size*; and iv) *peer influence*. The attendee group stated that too much sprinting/running, peer behaviour (e.g., disruptive individuals) and peer isolation negatively influenced their experience, whereas those in the non-attendee group only raised a lack of enjoyment and peer group (e.g., lack of friends’ attendance) as important influential factors.

#### Game features of the session

Game features of the exercise sessions were identified as the greatest facilitator, with the majority of the attendee group (81%) and non-attendee group (52%) outlining that games motivated them to participate and were what they enjoyed: “*the games mainly, because like they were fun*” (A:B12) and “*the games at the end I really enjoyed*” (NA:B44).

#### Enjoyment experienced during the session

Enjoyment from participation was the next most common facilitator in both groups. Specifically, of the attendee group, 72% described enjoying the sessions: “*I thought it was really good and I couldn’t wait for the next one*” (A:G11), while 48% of the non-attendee group enjoyed the sessions: “*I enjoyed it and I would come again, I just wish I went more*” (NA:G34). Nonetheless, 32% of the non-attendee group did not enjoy the sessions: “*I didn’t like it… I just didn’t want to go*” (NA:B39).

#### Competitive aspects of the session

Over half of the attendee group (53%) highlighted that the competition aspects of the sessions encouraged their participation: “*because you took part… it made it more competitive and enjoyed a lot more*” (A:B13). However, this was only the case for 18% of the non-attendee group.

#### Challenging nature of the exercise

An element which facilitated participation was the challenge of the exercises; a third (33%) of the attendee group suggested this and that they enjoyed pushing themselves: “*it was good to like get pushed, but it was hard as well, so it was good to like reach your limit, so you benefit*” (A:B22). However, the challenging nature of the session was also seen as a barrier, with the majority of the attendee group (60%) suggesting that the sessions were too challenging and that they were pushed too hard: “*It was pushing us over our limits, so it was too hard*” (A:B21). This contrast was also found among the non-attendee group; only 11% liked the challenge: “*trying to push yourself with challenges*” (NA:B29), while 16% found it a barrier for participating: “*I didn’t go… because it was hard*” (NA:G29).

#### The variation of the session content

Whilst 31% of the attendee group suggested that the variation of the session content was a facilitating factor: “*I really liked when we didn’t know what exercise was coming up next*” (A:B9), a greater proportion (47%) suggested that the session content was overly repetitive and lacked variation: “*for me it started off good, but then it started going down because it was getting repetitive*” (A:B14). Variation and repetition were described by 14 and 20% of the non-attendee group, respectively, despite only attending < 5% of the sessions.

#### Peer influence

Amongst the additional barriers that were highlighted, peers were the main focus. The attendee group (75%) noted that disruptive behaviour by other participants during the exercise sessions discouraged further participation: “*I didn’t enjoy the messing about because people have actually chosen to do it and if you have people messing around its really annoying*” (A:G3). However, being removed from their peer group (peer isolation) during a session due to disruptive behaviour was perceived negatively by 33% of the attendee group, despite being used as a strategy to control disruptive behaviour. Of the non-attendee group, 14% stated that their close friends not attending the sessions was a barrier. They also raised an aversion to exercising with other year groups, with over a quarter (27%) not wanting to exercise with either the older or younger year groups: “*It was intimidating [older year groups]*” (NA:G23).

### Intervention instructor

Both groups identified three instructor-related facilitators to attending the intervention: i) *their demeanour*; ii) *discipline*; and iii) *motivational abilities*. However, the attendee group also expressed three instructor-related barriers to participation: i) *their demeanour*; ii) *coaching ability*; and iii) *favouritism*. The instructor, specifically, was raised as a barrier by 16% of the non-attendee group.

#### Instructor demeanour

The instructors’ demeanour was viewed as a facilitator by a third of the attendee group (33%) and the non-attendee group (34%). An attendee highlighted: “*you would just enjoy it and it was just easy to go to if like you needed to say anything to him*” (A:B13), whilst a non-attendee stated: “*he like always makes everyone laugh so you’re in a happy mood to do energetic stuff*” (NA:G34). Despite the positive opinions, half of the attendee group (50%) found the demeanour of the instructor to be a barrier to attending the intervention and noted the instructor was “*a bit too angry*” (A:B9). More favourably, 31% of the attendee group and 23% of the non-attendee group found the instructor’s motivational abilities as a facilitating factor as they highlighted the instructor “*pushed us to our limits so we worked better*” (A:B17) and “*works you to the best of your ability*” (NA:G23).

Only 11% of the non-attendee group highlighted the instructor’s disciplinary approach as a facilitator, which more than doubled amongst the attendee group (25%). For example, *“I liked him for being strict sometimes because otherwise I wouldn’t have done anything*” (A:G7). However, over a third of the attendee group (36%) questioned the instructors coaching ability and felt the instructor did not provide any verbal reinforcement for the class’s participation. In addition, 19% felt aggrieved that the instructor favoured the older year groups, *“He would just focus on like the older kids and how good they would be doing*” (A:B20).

### Additional barriers

The *timings of the sessions* and *competing interests* were highlighted as additional key barriers to long-term adherence. Half of the attendee group (50%) felt the morning sessions were too early and that they were too exhausted to attend the sessions after school, and 42% identified competing interests, such as paper rounds, school work or music lessons, prevented their attendance: “*I just couldn’t handle the school work and the exercise*” (A:B17). Despite only attending a small proportion of the sessions, similar barriers were relayed among the non-attendee group for timing of the sessions (41%) and competing interests (48%): “*I didn’t have time because before school I have to do a paper round… well I have football training and rugby training, so I just can’t fit it in really*” (NA:B40). An additional barrier highlighted only by the non-attendee group was a lack of motivation (27%): *“I purely couldn’t be bothered… it’s too early in the morning… and I just couldn’t be bothered to go after school*” (NA:G28).

## Discussion

This study sought to identify adolescents’ perceptions and reflections of participating in a 6-month HIIT intervention, delivered either before or after school, and to identify key facilitators and barriers for consideration in the design of future interventions. This work adds to an evolving evidence base on adolescents’ perceptions of HIIT interventions and, more specifically, to the under-explored area of HIIT interventions in school settings. Findings show that a HIIT intervention can be an enjoyable form of exercise, however, irrespective of participant attendance, consistent facilitators and barriers to participating in the intervention, such as session timing, content of the exercise session, group composition, instructor characteristics and exercise difficulty, were identified. Overall, intervention adherence was low in comparison to other school-based HIIT interventions where, on average, participants attended 2 out of 3 sessions a week over 7–14 weeks [12, 19, 20]. It is possible that the longer duration of each exercise session in our X4ACJ programme, and the overall longer intervention duration (six months), compared to other school-based HIIT interventions may, at least in part, explain the lack of sustained adherence. Further work is required to elucidate the minimum overall dose and frequency for such school-based interventions to elicit meaningful enhancements in key health outcomes whilst maximising sustained attendance.

The primary facilitator to participation in HIIT was the perceived personal benefits of exercise, particularly physiological improvements, which were identified by at least two thirds of the participants, irrespective of group. In line with Leahy et al. [12], the perceived impact on overall health was identified as the main physiological benefit. However, whilst Leahy et al. [12] reported that participants were most motivated to attend to improve their health [12], not all adolescents have previously recognised the important connection between physical activity and health [31, 32]. Given the increased campaigns highlighting the health benefits of physical activity over the past decade, adolescents may now have a greater awareness. However, awareness and participation do not always align [33]. For example, despite around a third (34%) of the non-attendees in this study acknowledging the physiological benefits, they attended less than 5% of the sessions available to them. Consistent with previous research [33], awareness of physical activity benefits, in isolation, is not sufficient to translate to actual behaviour change. This emphasises the importance of understanding what motivates individuals to be physically active. For example, in accord with the self-determination theory [34], feedback from health measures, such as changes in body composition or fitness, may facilitate greater participation for extrinsically motivated (e.g., driven by external rewards) individuals [35]. In contrast, factors concerning competence and enjoyment are rated more highly as reasons for participation among those who are intrinsically motivated (e.g., driven by internal rewards [36]).

The content of the HIIT intervention exercise sessions was highlighted as both a facilitator and barrier to participation. Perceived enjoyment of exercise is a pivotal influence on physical activity adherence [37], and very few studies have explored adolescents’ enjoyment of HIIT [38]. This study found that the majority of the attendee group (72%) enjoyed participating in the intervention, which could explain their continued participation, while only 48% of the non-attendees commented that they enjoyed it. Social learning theory [39] suggests that many of an individual’s enjoyed behaviours could previously have been something they were averse to until they learnt the positive consequences, such as a sense of achievement. Moreover, 32% of the non-attendees talked about not enjoying the HIIT exercise sessions, a dominating barrier which was not discussed among adolescents who regularly attended. Enjoyment has been identified as a factor of intrinsic motivation, which in turn has been positively associated with physical activity levels [40]. It could be suggested that as the non-attendees participated in such a limited number of sessions, they did not provide themselves sufficient time to learn to enjoy the high-intensity nature of the exercise.

This study reinforces previous literature [10, 27, 38] that exercise sessions must be enjoyable to sustain participation. One method of delivering HIIT is through games, which has previously achieved an average attendance of 77% for a 10-week intervention [41]. The games aspect of the X4ACJ programme, which was implemented in one of three sessions a week, was viewed as a facilitator to participation in both groups. However, it is unknown whether games would be viewed as favourably among adolescents if they were the sole method of the HIIT intervention, as has been found among young children [42]. Further research is therefore required to determine whether an increase in game content would increase participant retention and elicit similarly advantageous responses as the increased cardiorespiratory fitness and maintained body mass index observed in the X4ACJ programme [22]. Finally, HIIT has been perceived as difficult by adolescents in the formative research for the X4ACJ programme [43], and as not suitable for the general population due to the discomfort participants experience [12]. Whilst those in the current study reflected that the X4ACJ programme was challenging, this was reported in both groups and more commonly in those who attended, suggesting that this did not serve as a sufficient barrier to prevent involvement in the sessions.

In addition to the content of the X4ACJ programme being a facilitator and a barrier, there were conflicting perceptions of the intervention instructor. The effects of modelling on physical activity in adolescents has been well explored, with positive role modelling increasing physical activity levels [44, 45] and poor role modelling of not engaging in physical activity linked to low levels of physical activity [46]. Twice as many adolescents in the attendee group highlighted the instructor’s disciplinary approach (e.g., being strict which kept participants on task) as a facilitator as compared to the non-attendee group, suggesting the approach could have encouraged attendance. However, the disparity between participants’ perceptions of the instructor is due to the instructor having to discipline misbehaving participants. This may have given the impression of a negative demeanour and favouritism towards certain groups. Costigan et al. [19] suggested that if an instructor delivers HIIT using an authoritarian teaching style (i.e., dictating firm realistic boundaries), it could result in a lack of enjoyment. As such, this highlights the significant role instructors can have on influencing the behaviour of individuals. Previous school-based HIIT interventions have been delivered by qualified teachers [12, 19], or have been delivered with the support of qualified teachers [20], therefore this study provides a new approach of an ex-military individual delivering the session who was external to the school and has less regular contact with adolescents. In addition, verbal behaviour, the words used with the function of motivating individuals, can impact physical activity performance [47]. In the current study, both groups reflected on the verbal behaviour of the intervention instructor as a barrier to participating, highlighting that instructors should be skilled at working with adolescents, as well as skilled at delivering the exercise content in order to minimise a preventable barrier.

Whilst many of barriers to the intervention were somewhat unsurprising, the identification of sprinting/running exercises as a barrier was unexpected given that these were specifically incorporated based on formative research and co-design with adolescents [43]. These findings therefore question the reliability of adolescents’ pre-conceived perceptions of exercise and, indeed, the application of the Theory of Planned Behaviour [15], given that the participants’ perceptions did not match their behaviour. Incorporating “taster” sessions within traditional formative research methods may lead to more informed perceptions among the target population and better co-designed interventions.

It is important that external factors are considered for future design and implementation of HIIT interventions. Between 40 and 50% of participants in both groups highlighted that the time of the programme delivery was a barrier and that they had competing interests (e.g., recreational activities, including sport, or jobs). As such, future interventions should consider moulding their interventions to the school curriculum for physical education or use other “available” slots throughout the school day, such as registration or breaktime, to increase participation. Interventions must be flexible in their design and accommodate competing interests.

This study has several strengths, including a balanced number of boys and girls within the attending and non-attending groups, providing a sex-balanced perspective of the intervention. In addition, the use of a non-attendee group provided insight to the study which would have not been highlighted otherwise. Nonetheless, certain limitations must be acknowledged. It is important to consider the potential influence of self-selection bias on the present findings due to the voluntary nature of the intervention [48]. In addition, the participants attended a school in a more affluent urban area; adolescents in more deprived areas may not share the same facilitators and barriers to participation and thus findings from one school may not be generalisable to other schools. Whilst the present study included participants who signed up to the intervention but did not regularly attend, it could be argued that these participants were still biased, given they contemplated participation. An additional limitation is that peer pressure may have influenced focus group discussions and therefore outcomes. Specifically, in order to conform with perceived socially-desirable responses, participants may have modelled their answers accordingly [49]. Participants may also have felt pressured not to oppose, or alternatively, to agree with dominant members of the group [50], or withhold their opinions. Whilst the author conducting the focus groups had attended the exercise sessions and assessment days and built a rapport with the attendee and non-attendee participants to promote extraction of more insightful information, participants may not have felt comfortable being critical of the intervention. An additional limitation to the study was the lack of interviews with other people involved with the intervention, such as the Commando Joe’s instructor. Eliciting the opinions of the HIIT intervention from a facilitator’s standpoint may have provided an alternative, yet valuable, insight into some of the themes extracted from the attendee and non-attendee participants.

## Conclusion

This study adds to a sparse body of literature exploring adolescents’ perceptions of school-based HIIT programmes and barriers and facilitators to participation. Overall, our findings demonstrated that HIIT can be an enjoyable form of exercise and can be implemented in a school setting. However, there is a need to be flexible and look to incorporate exercise programmes into the school day, as timing of the sessions were found to be a barrier. Furthermore, it important to educate adolescents on the possible benefits of HIIT interventions and making the sessions as enjoyable as possible to increase their motivation to participate. These findings are important to consider for future public health interventions targeting adolescents.

## Data Availability

The datasets used during the current study are available from the corresponding author on reasonable request.

## Abbreviations

HIIT: High intensity interval training
YPAMA: Youth Physical Activity Promotion Model
WIMD: Welsh Index of Multiple Deprivation
A: Attendee group
NA: Non-attendee group
WTBE: William T B Eddolls
KAM: Kelly A Mackintosh
MAM: Melitta A McNarry
B: Boy
G: Girl
